# Preparation and Characterization of Optimized Hydrochar from Paper Board Mill Sludge

**DOI:** 10.1038/s41598-019-57163-7

**Published:** 2020-01-21

**Authors:** Sadish Oumabady, Paul Sebastian S., Sara P. B. Kamaludeen, Mahendiran Ramasamy, P. Kalaiselvi, E. Parameswari

**Affiliations:** 1 Department of Environmental Sciences, Tamil Nadu Agricultural University, Coimbatore, 641 003 India; 2 Department of Renewable Energy and Engineering, Tamil Nadu Agricultural University, Coimbatore, 641 003 India

**Keywords:** Environmental sciences, Energy science and technology

## Abstract

The amount of Paper Board Mill Effluent Treatment Plant Sludge (PBM-ETPS) dumped from paper mills are huge and its conversion into hydrochar for the purpose of energy has broad prospects. This study investigated the optimum conditions for the production of PBM-ETPS derived hydrochar (PBM-ETPSH) through Reponse Surface Methodology (RSM) for more surface area and pore volume with minimal hydrogen to carbon (H/C) and oxygen to carbon (O/C) ratios. The PBM-ETPSH had higher heating value (HHV) of 18.39 MJ kg^−1^ with fixed carbon percentage of 15.6. Our results showed a reduction in H/C (35.05%) and O/C (43.7%) ratios confirming the coalification of optimized PBM-ETPSH. Thermogravimetric investigations of blending PBM-ETPSH with coal in 1:1 ratio increased its HHV to 22.25 MJ kg^−1^ making it suitable as an energy alternative for paper mills.

## Introduction

The advent of industrial revolution has boosted an enormous generation of liquid and solid wastes leading to advanced treatment technologies. Hydrothermal carbonization (HTC) is an emerging method for the conversion of wet biomass at relatively mild reaction temperatures into a coal-like material called as hydrochar^1^. Technically, the conversion process was performed in the temperature range of 180 °C–260 °C for 30 minutes to 10 h with biomass to water ratio of 1:9^2^. The HTC process is governed by different process parameters such as temperature, time, biomass to water ratio, pH of the substrate and concentration of the catalyst, among which the temperature and time are significant. Higher temperature promoted lower solid yield production with higher quantity of gaseous compounds^3^. In another study, the increase of HTC temperature from 180 °C to 300 °C has resulted in the solid yield reduction of hydrochar from 66.18% to 53.00%^4^. The distribution and quality of solid, liquid and gaseous products are influenced by the residence time of the reaction and 12 h residence time resulted 60% increase in the solid yield of hydrochar per unit biomass due to the polymerization of fragments in the dissolved phase thereby forming secondary hydrochar with a polyaromatic structure^5^.

Being a carbon compound with good aromaticity, hydrochar has a potential application in the energy sector as an alternative source of energy due to the reduction of inorganic constituents and increased proportions of carbon compounds^6^. Thus, conventional polluting energy sources can be replaced by hydrothermally carbonized products thereby ensuring sustainability. The thermal behavior of hydrochar was found similar to the non-renewable energy sources such as coal, lignite and anthracite thus reducing the dependency on conventional energy sources^1^. Hydrochar also possess more calorific value than any other carbon based material due to complete removal of volatile matter with an increase in the ash content thereby resulting in higher heat generation during combustion^7^. Hence the hydrochar can be used as a coal substitute for heat production through co-combustion process at specific proportions.

The global production of paper accounted for about 413 million tons in 2017 of which India contributed 3.18% through paper, paperboard and newsprint production per annum^8^. The paper products were recycled after utilization through paper board mill industries thereby producing recycled paper and packaging materials with PBM-ETPS as the predominant solid waste. Since, the present study was taken to convert the PBM-ETPS into Hydrochar by HTC process, the optimization of process parameters are considered to be an important for producing hydrochar from PBM-ETPS with anticipated surface and fuel characteristics which was made ease by employing statistical tool named Response Surface Methodology (RSM). Investigations on thermal behavior of the hydrochar also paved way for its utilization as an alternate energy source.

Based on this scenario, the current research was performed on the (a) optimization of process temperature and time for hydrochar production from PBM-ETPS using RSM, (b) characterization of hydrochar in terms of proximate and elemental composition, structural morphologies, surface functionalities and textural properties. Additionally, the higher heating value and thermogravimetric investigations of the optimized hydrochar and commercially utilized coal at different blends were carried out to appraise the combustion behavior for environmental applications.

## Experimental Methods

### Paper board mill-effluent treatment plant sludge

The PBM-ETPS was collected from Effluent Treatment Plant (ETP) at ITC Ltd., PSPD (Kovai unit), Coimbatore, India and stored in sample containers at 4 °C. PBM-ETPS was oven dried at 105 °C for moisture removal and ground, sieved through 2 mm sieve, subsequently stored to perform the initial characterization^1^.

### Hydrothermal carbonization

**Figure 1:**
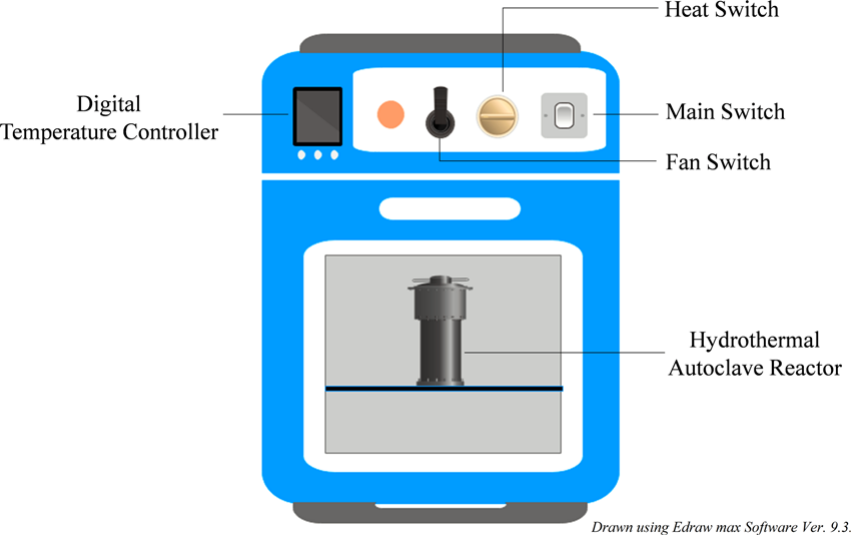
Schematic diagram of experimental Setup.

The PBM-ETPS was mixed before the experiment for attaining homogeneity. After homogenization, 60 g of fresh PBM-ETPS (containing 1:9 sludge:water proportion) was taken in a 100 ml hydrothermal autoclave reactor and sealed^1^. In order to achieve oxygen free conditions, nitrogen gas was purged into the reactor for 2 minutes^9^. The reactor was then placed in the laboratory oven (Fig. 1) and heated to the desired temperatures for different reaction times. After the completion of reaction, the reactor was quickly moved from the oven and submerged in the cold water to quench the reaction^2^. The formed hydrochar (PBM-ETPSH) was filtered in order to separate the solid and liquid portion and was allowed to dry overnight at 105 °C in hot air oven followed by storing in an air tight container.

### Characterization of PBM-ETPS and PBM-ETPSH

The pH and Electrical Conductivity of the samples were determined at a solid to water suspension ratio of 1:2.5 using digital pH meter and conductivity meter, respectively. The heavy metal content (Cd, Cr, Ni and Pb) of the acid digested samples were determined in Atomic absorption spectrophotometer (Perkin Elmer AA analyst 400). The proximate characterization of PBM-ETPS and PBM-ETPSH were carried out by standard ASTM procedures (ASTM-E871-82, ASTM-D3175-07 and ASTM-D3174-04). The ultimate composition (C, H, N, S and O) was determined by Elementar vario EL III. The BET Surface area and pore volume were determined by smartsorb 92/93 surface area analyzer. The surface morphologies and texture characteristics of the samples were interpreted by scanning electron microscope M/s. FEI - Quanta 250, Czech Republic taken at a high voltage of 8 kV with 10,000x magnification and transmission electron microscope M/s. FEI - Quanta 250, Czech Republic operating at an accelerating voltage of 120 kV^10^. The functional groups were determined using FTIR (Model 8400 S of Shimadzu, Japan) over the wavenumber range of 400–4000 cm^−1^ ^11^. The particle size and zeta potential were measured using the particle size analyzer, Horiba Scientific Nanopartica SZ-100, Japan^12^.

### Optimization of PBM-ETPSH production

The Integrated optimal (I-optimal) design of response surface methodology was employed for the hydrothermal carbonization of PBM-ETPS. The I-optimal design was chosen to match a quadratic polynomial model with least number of experiments which facilitated the investigation of interaction between the process parameters and recognized the main factor for response optimization. The process parameters for optimization and their respective coded levels are listed in the Table 1. BET surface area, pore volume, hydrogen to carbon (H/C) ratio and oxygen to carbon (O/C) ratio were set as target parameters (responses) for the optimization process. Sixteen runs of the experiments were randomly suggested by the experimental design for the production of PBM-ETPSH. The randomized runs of the experiments and their responses are listed in the Table 2. The response of every run was analyzed using design expert software version 11 from Stat-Ease Inc. 2000. It led to the optimization of process parameters (temperature and time) for achieving desired target parameters such as maximum surface area, maximum pore volume, minimum H/C ratio and minimum O/C ratio. The analysis of variance (ANOVA) was carried out to analyze the experimental results as shown in the Table 3. The probability (p-value) and Fischer test value (F-value) were used to compute the regression model for all the responses. The model of all the responses were found to be significant due to higher F-value and lower p-value. The best fitted model for the BET surface area, pore volume, H/C ratio was quadratic model with a R^2^ value of 0.7706, 0.8812 and 0.8607, respectively, while the best fitted model for O/C ratio was 2 factor interaction (2FI) model with a R^2^ of 0.7524. The corresponding equations for the each responses were,

1 $${{\rm{Y}}}_{1}({\rm{BET}}\,{\rm{Surface}}\,{\rm{area}})=5.83+0.1846{\rm{A}}+0.5953{\rm{B}}-0.3807{\rm{AB}}-2.93{{\rm{A}}}^{2}+0.6595{{\rm{B}}}^{2}$$

2 $${{\rm{Y}}}_{2}({\rm{Pore}}\,{\rm{volume}})=0.0083-0.0013{\rm{A}}+0.0004{\rm{B}}-0.0015{\rm{AB}}-0.0038{{\rm{A}}}^{2}+0.0009{{\rm{B}}}^{2}$$

3 $${{\rm{Y}}}_{3}({\rm{H}}/{\rm{C}}\,{\rm{ratio}})=1.23-0.0747{\rm{A}}-0.1135{\rm{B}}-0.0511{\rm{AB}}+0.0995{{\rm{A}}}^{2}-0.0011{{\rm{B}}}^{2}$$

4 $${{\rm{Y}}}_{4}({\rm{O}}/{\rm{C}}\,{\rm{ratio}})=0.4942-0.0381{\rm{A}}-0.0673{\rm{B}}-0.0196{\rm{AB}}$$

where, A denotes the coded value of temperature and B shows the coded value of time. The effect of specific factor and the interaction among the factors were pointed out by coefficient with one and two factor respectively wherein a positive sign denotes synergistic relation while a negative sign denotes antagonistic relation.

**Table 1:** Code levels of variables for I-optimal design (RSM tool).

Factor Name	Code levels of process parameters			
	Level I	Level II	Level III	Level IV
Temperature (°C)	180	200	220	240
Time (h)	4	6	8	10

**Table 2:** Experimental design runs and their analogous results (RSM tool).

Run	Temperature (°C)	Time (h)	BET Surface Area (m^2^ g^−1^)	Pore Volume (cc g^−1^)	H/C ratio	O/C ratio
1	200	8	5.92	0.0096	1.25	0.48
2	240	4	3.99	0.0052	1.40	0.59
3	240	6	2.85	0.0036	1.32	0.50
4	200	8	4.12	0.0067	1.24	0.48
5	200	4	5.24	0.0081	1.34	0.55
6	180	6	3.58	0.0068	1.52	0.60
7	220	6	6.06	0.0082	1.20	0.51
8	240	6	1.93	0.0030	1.31	0.49
9	200	8	5.93	0.0086	1.25	0.48
10	220	4	5.79	0.0085	1.41	0.45
11	220	6	6.31	0.0083	1.20	0.53
12	180	10	4.29	0.0092	1.33	0.49
13	180	8	3.27	0.0056	1.32	0.49
14	180	4	1.65	0.0042	1.41	0.60
15	240	10	4.32	0.0030	1.09	0.36
16	240	10	4.03	0.0029	1.09	0.37

(Samples analyzed under moisture free basis and the values represent average of triplicate)

**Table 3:** ANOVA for surface area, pore volume, H/C ratio and O/C ratio.

Source	Surface area			Pore volume			H/C ratio			O/C ratio		
	F-value	P-value	S/NS	F-value	P-value	S/NS	F-value	P-value	S/NS	F-value	P-value	S/NS
Model	6.72	0.005	S	14.83	0.0002	S	12.36	0.0005	S	12.15	0.0006	S
A-temperature	0.44	0.520	NS	16.70	0.0022	S	18.62	0.0015	S	9.55	0.0093	S
B-time	3.50	0.090	NS	1.01	0.3380	NS	32.68	0.0002	S	23.65	0.0004	S
AB	1.04	0.331	NS	12.02	0.0060	S	4.81	0.0530	NS	1.46	0.2500	NS
A²	31.93	0.0002	S	37.85	0.0001	S	9.42	0.0118	S			
B²	1.57	0.238	NS	2.02	0.1858	NS	0.001	0.9737	NS			
Lack of Fit	1.74	0.279	NS	1.26	0.4028	NS	790.9	0.0001	S	30.25	0.0008	S

(S – Significant, NS – Non-significant)

### Fuel properties of PBM-ETPSH and its blending with coal

PBM-ETPSH was blended with commercial coal at different percentages i.e., 0, 10, 20, 30, 40 and 50% and characterized for its fuel properties. The higher heating value (HHV) of the samples were determined using digital bomb calorimeter. The thermal stability and the combustion behavior of the samples were assessed through simultaneous TGA/DSC analysis using Perkin Elmer simultaneous thermal analyzer (STA 6000). It operates under the working temperature of 0–900 °C under argon gas to ensure inert atmosphere with a heating rate of 5 °C min^−1^ ^13^. Based on the HHVs, the economic feasibility of the hydrothermal process was assessed by different parameters like Hydrochar yield, Energy densification and Energetic recovery efficiency^1^.

$${\rm{Hydrochar}}\,{\rm{yield}}( \% )=({\rm{Mass}}\,{\rm{of}}\,{\rm{Hydrochar}}/{\rm{Mass}}\,{\rm{of}}\,{\rm{feedstock}})\times 100$$

$${\rm{Energy}}\,{\rm{Densification}}={\rm{HHV}}\,{\rm{of}}\,{\rm{Hydrochar}}/{\rm{HHV}}\,{\rm{of}}\,{\rm{feedstock}}$$

$${\rm{Energetic}}\,{\rm{recovery}}\,{\rm{efficiency}}\,( \% )={\rm{Hydrochar}}\,{\rm{yield}}\times {\rm{Energy}}\,{\rm{Densification}}$$

## Results and Discussion

### Characterization of PBM-ETPS

The physico-chemical, proximate and ultimate characterization of PBM-ETPS are listed in the Table 4. The pH of the PBM-ETPS was 6.36 with the electrical conductivity of 5.36 dSm^−1^. The proximate characterization of PBM-ETPS showed a volatile matter content of 62.5%, ash content of 30% and fixed carbon content of 7.4%. The ultimate characterization of PBM-ETPS showed a carbon content of 29.69%, hydrogen content of 4.34%, nitrogen content of 3.28%, sulfur content of 1.04% and oxygen content of 31.65%.The HHV of PBM-ETPS was 17.15 MJ kg^−1^. The BET surface area and pore volume of PBM-ETP sludge were 0.75 m^2^ g^−1^ and 0.001 cc g^−1^, respectively. The particle size of the PBM-ETP sludge was 277.5 nm while its zeta potential was −16.4 mV. Among the heavy metals, the lead (Pb) content was 82 mg kg^−1^, while chromium, cadmium and nickel were below detectable limit (BDL).

**Table 4:** Characterization of PBM-ETPS, PBM-ETPSH and Commercial Coal.

Parameter	PBM-ETPS	PBM-ETPSH	Commercial Coal^24^
pH	6.36	5.79	—
EC (dS m^−1^)	5.36	6.99	—
Higher Heating Value (MJ kg^−1^)	17.16	18.39	11–30
**Proximate Analysis (%)**			
Volatile matter	62.5	44.5	16–30
Ash content	30	39.9	25–50
Fixed carbon	7.4	15.6	24–40
**Ultimate Analysis (%)**			
Carbon	29.69	32.77	30–55
Hydrogen	4.34	3.13	2–4
Nitrogen	3.28	3.32	0.7–1.15
Sulfur	1.04	0.82	0.3–0.8
Oxygen	31.65	20.07	4–8
H/C ratio	1.74	1.13	—
O/C ratio	0.80	0.45	—
**Heavy metals (mg kg**^**−1**^**)**			
Chromium (Cr)	bdl	bdl	—
Cadmium (Cd)	bdl	bdl	—
Lead (Pb)	82	bdl	—
Nickel (Ni)	bdl	bdl	—

(Samples were analysed on dry basis and the values represent average of triplicates)

### Process optimization of hydrochar production

**Figure 2:**
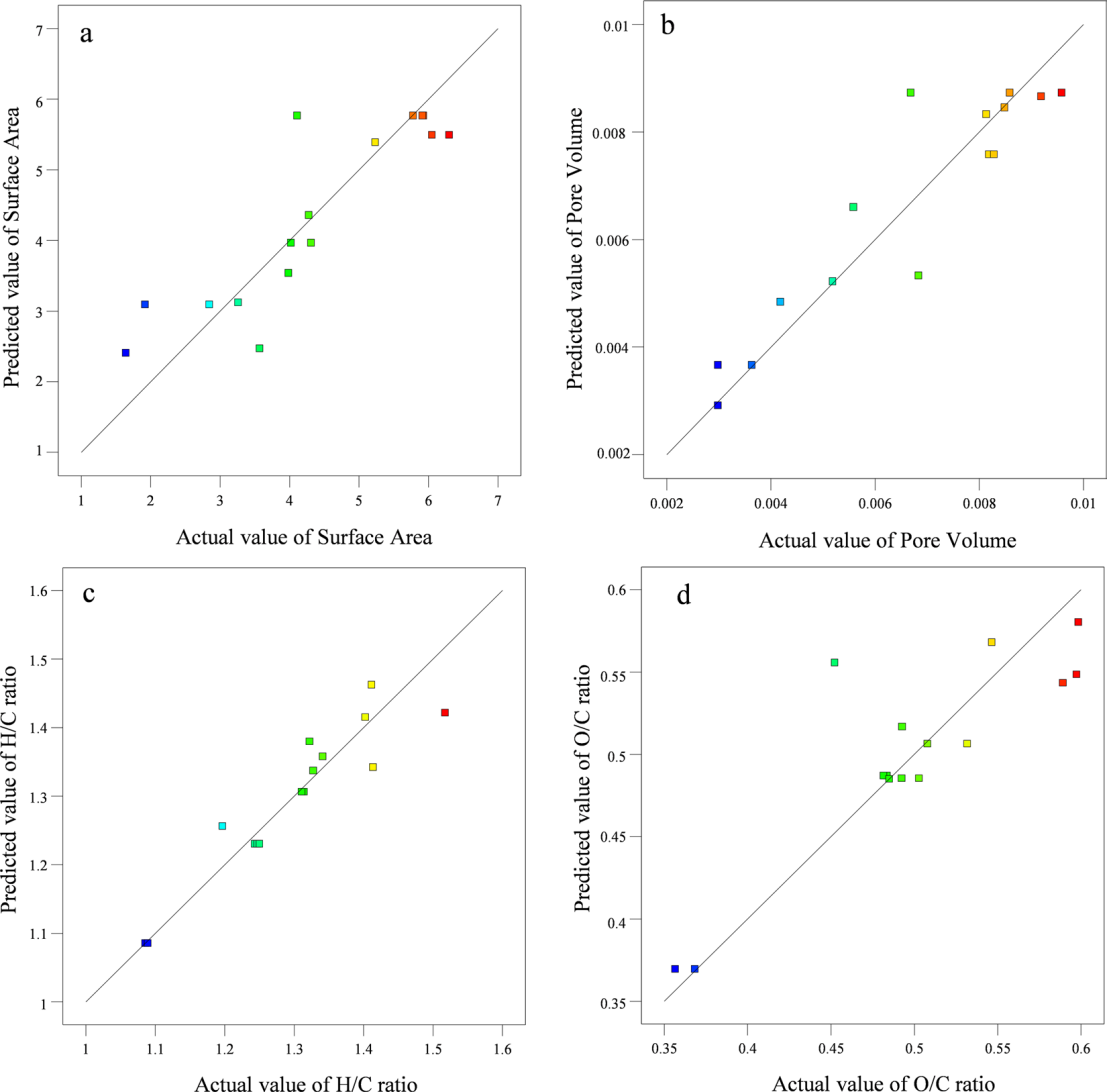
Relationship between actual and predicted values of (**a**) BET surface area, (**b**) Pore volume, (**c**) H/C ratio and (**d)**. O/C ratio.

**Figure 3:**
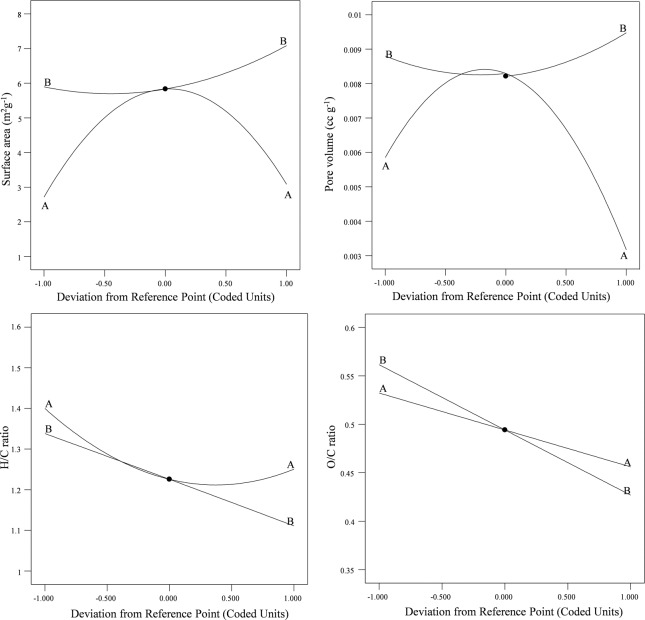
Effect of perturbation plot on individual responses. (**a**) BET surface area, (**b**) Pore volume, (**c**) H/C ratio and (**d**) O/C ratio.

**Figure 4:**
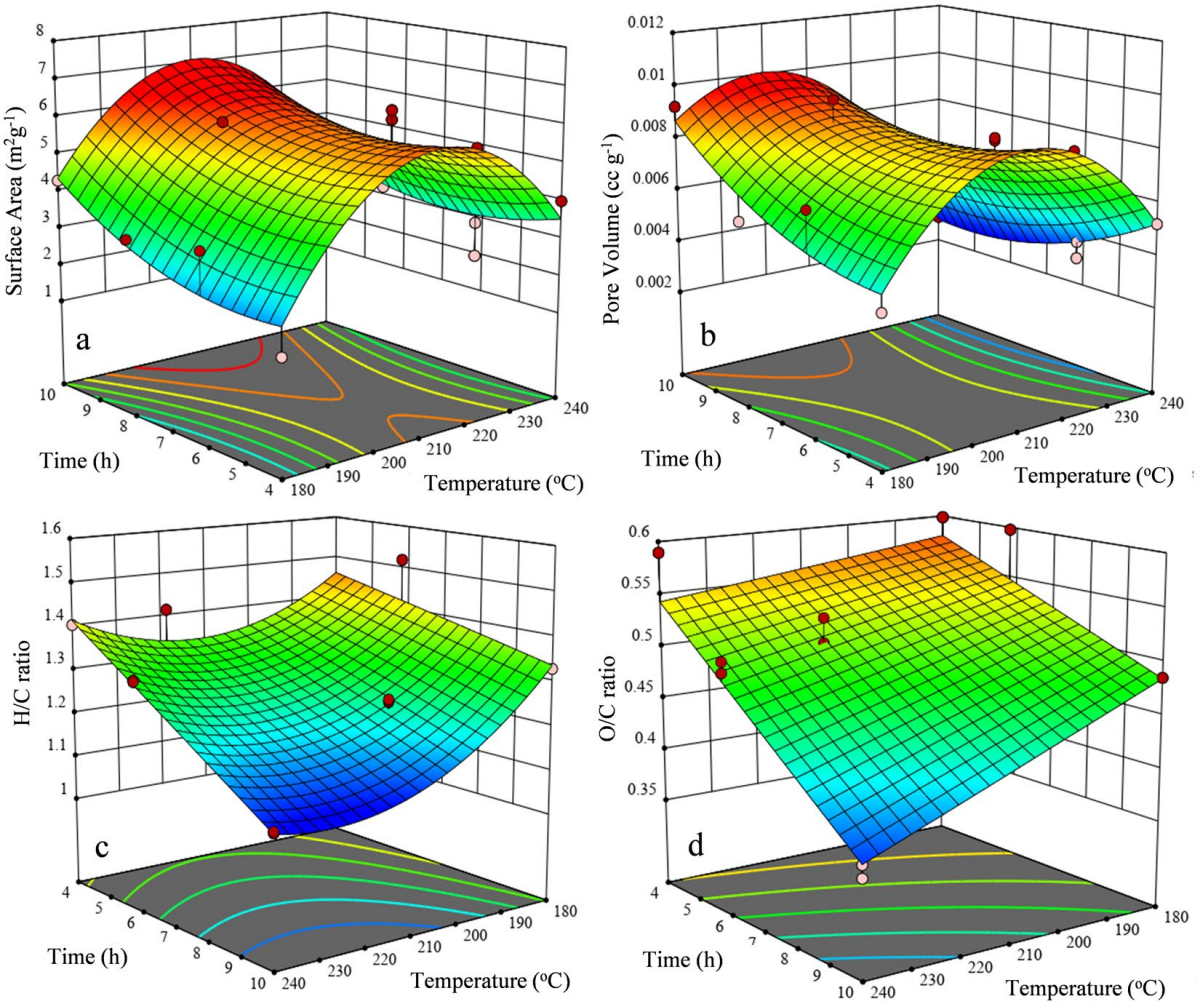
Three-dimensional response surface curve of temperature and time on (**a**). Surface Area, (**b**) Pore Volume, (**c**) H/C ratio and (**d**). O/C ratio.

The plotted graph exhibiting predicted versus experimental values of the every responses are given in the Fig. 2. The experimental values of the responses were found closer to the predicted values which revealed that the developed model showed a good correlation between the process parameters and its responses. The effect of process parameters and their interaction on the production of PBM-ETPSH were determined using perturbation plots (Fig. 3) and three dimensional response surface plots (Fig. 4). The perturbation plots helps in evaluating the effect of all process parameter at a specific point and monitoring its behavior to check the changes undergone by every response for respective change in process parameter^14^. The extent of sensitiveness of BET surface area, pore volume and H/C ratio to the process parameters were highlighted by a steep slope or a curvature, while the impervious nature of O/C ratio to the process parameters was denoted by a flat line. In case of three dimensional plots, red region denotes the higher value of responses while the blue region denotes the lower value of responses. It was noticed that the surface area and pore volume correspondingly increased with the increase in process temperature and time, while H/C and O/C ratios decreased eventually. The statistical optimization has resulted in the projection of some solutions (Optimized process parameters) among which, the process temperature of 200 °C and time of 10 h was concluded to be the optimized process parameter for the production of PBM-ETPSH due to its highest predicted probability of 84%. The optimized PBM-ETPSH may possess a surface area of 6.8 m^2^ g^−1^, pore volume of 0.010 cc g^−1^, H/C ratio of 1.164 and O/C ratio of 0.446.

### Characterization of PBM-ETPSH

#### Physico-chemical, proximate and ultimate characterization

The physico-chemical, proximate and ultimate characterization of PBM-ETPSH at 200°C for 10 h are listed in the Table 4. The pH of the optimized PBM-ETPSH (5.79) was acidic than its precursor i.e PBM-ETPS (6.36). This can be substantiated by the formation of some acidic groups like acetic acid, formic acid, glycolic acid, levulinic acid that were formed due to the degradation of organic compounds^15^. The measured electrical conductivity of PBM-ETPSH (6.99 dS m^−1^) was more than its precursor PBM-ETPS (5.36 dS m^−1^). The increased electrical conductivity was due to the adsorption of dissociated salts from the raw material during hydrothermal carbonization^16^. The lead content of the PBM-ETPS diminished after hydrothermal carbonization from 82 mg kg^−1^ to below detection limit in PBM-ETPSH which might be due to the leaching of lead from solid to liquid portions. The proximate investigation showed that the volatile matter content decreased by 18% due to chemical dehydration and decarboxylation reactions while, the fixed carbon percentage increased by 8.2% due to reduction in volatile matter and simultaneous aromatization accompanied with repolymerization reactions^17^. Nevertheless, the increment in fixed carbon (8.2%) was lower than the loss in volatile matter (18%) thereby indicating conversion of volatile compounds into liquid and gaseous products, respectively.

**Figure 5:**
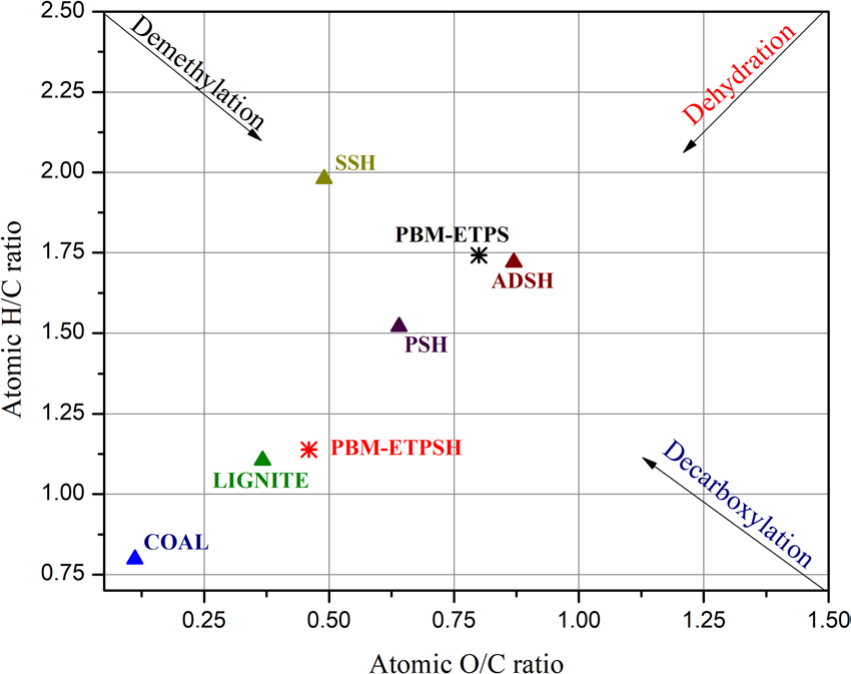
Van Krevelen diagram for assessment of coalification degree.

The process of HTC resulted in the increase of carbon content by 3.08% while, it decreased the hydrogen and oxygen by 1.21% and 11.58%, respectively. This resulted in the reduction of hydrogen to carbon (H/C) and oxygen to carbon (O/C) ratios from 1.74 and 0.80 to 1.13 and 0.45 for PBM-ETPS and PBM-ETPSH, respectively. The reduction was observed due to the prevalence of decarboxylation, dehydration and demethylation reactions during hydrothermal carbonization. The magnitude of these reactions were assessed by Van Krevelen diagram in which the H/C and O/C ratio of PBM-ETPSH was appraised with conventional energy products like coal and lignin thereby inspecting the degree of coalification. Generally, a compound with lower H/C and O/C ratio can be considered as a fuel^18^. The coalification degree of the PBM- ETPS, PBM-ETPSH and other related hydrochar compounds like sewage sludge derived hydrochar (SSH)^4^, paper sludge derived hydrochar (PSH)^6^, anaerobic digested sludge derived hydrochar (ADSH)^5^ were compared with commercial coal and lignite (Fig. 5).

#### Surface functionalities of PBM-ETPS and PBM-ETPSH

**Figure 6:**
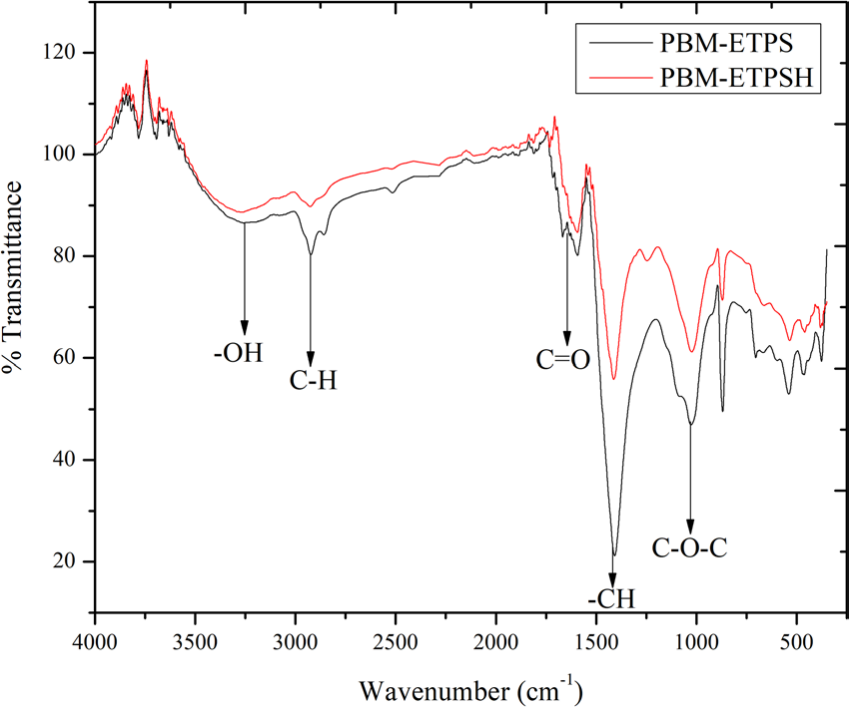
FTIR spectra of PBM-ETPS and PBM-ETPSH.

The FTIR spectra (Fig. 6) of PBM-ETPS and PBM-ETPSH depicted a broad band with variable stretching around 3200–3600 cm^−1^ which was due to the presence of cellulose in the PBM-ETPS. After HTC, OH stretching between 3200–3400 cm^−1^ has shown a gradual weakening which might be due to the prevalence of dehydration reactions^17^ and it can be correlated with the reduction of H/C and O/C ratios of PBM-ETPSH compared PBM-ETPS. Additionally, the reduction in hydroxyl groups led to the increase of hydrophobic, energy storage and facile handling properties thereby stating it as an efficient solid fuel^19^. A rounded tip band at 3260 cm^−1^ corresponded to the OH stretching of phenolic OH groups. The band around 2800 to 3000 cm^−1^ attributed to the vibration of aliphatic methyl groups wherein a centroid at 2920 cm^−1^ represented the vibration of asymmetric C–H stretching thereby indicating the presence of amino acids. The characteristic C=O stretching band at 1592 cm^−1^ corresponded to C–N stretching vibration of amides and the reduction was observed due to the decarboxylation reactions underwent during the hydrothermal carbonization process^20^. The band at 1412 cm^−1^ portrayed the presence of –CH aliphatic compounds like –CH_2_ and –CH_3_. The aliphatic –CH_x_ groups (1400–1500 cm^−1^) showed a sharp and narrow peaks in optimized PBM-ETPSH compared to the groups present in PBM-ETPS. This was due to the breaking up of polymeric substances during the hydrothermal carbonization process that resulted in the predominant appearance of aliphatic structures in PBM-ETPSH^6^. Irrespective of the raw biomass, the C=O stretching vibrations of ketone and amide has been decreased during the hydrothermal carbonization process thus resulting in the production of CO_2_ from the carbon precursors. The intensification of aliphatic bands at 1412 cm^−1^ portrays the release of aliphatic or non-aromatic compounds after the process and the tiny band at 1540 cm^−1^ corresponding to asymmetric C–O stretching has been eliminated at the end of the process due to decarboxylation reactions^5^. The peak corresponding to 1026 cm^−1^ depicted the asymmetric stretching of C–O–C because of dehydration reaction of alcohol.

#### Structural morphologies and textural characterization

The Brunauer-Emmett-Teller analysis of surface area depicted the increase in surface area and pore volume of optimized PBM-ETPSH from 0.75 m^2^g^−1^ to 3.74 m^2^ g^−1^ and 0.001 cc g^−1^ to 0.007 cc g^−1^, respectively. The particle size of the optimized PBM-ETPSH decreased from 277.5 nm to 104.7 nm. The protonation and deprotonation of polar functional groups like carboxyl and hydroxyl were responsible for the generation of zeta potential values. The decrease in zeta potential value of optimized PBM-ETPSH from −16.4 mV to −17.1 mV denoted the occurrence of decarboxylation and dehydration reactions. The negative zeta potential depicts the presence of negative charges on the surface of carbon material^5^.

**Figure 7:**
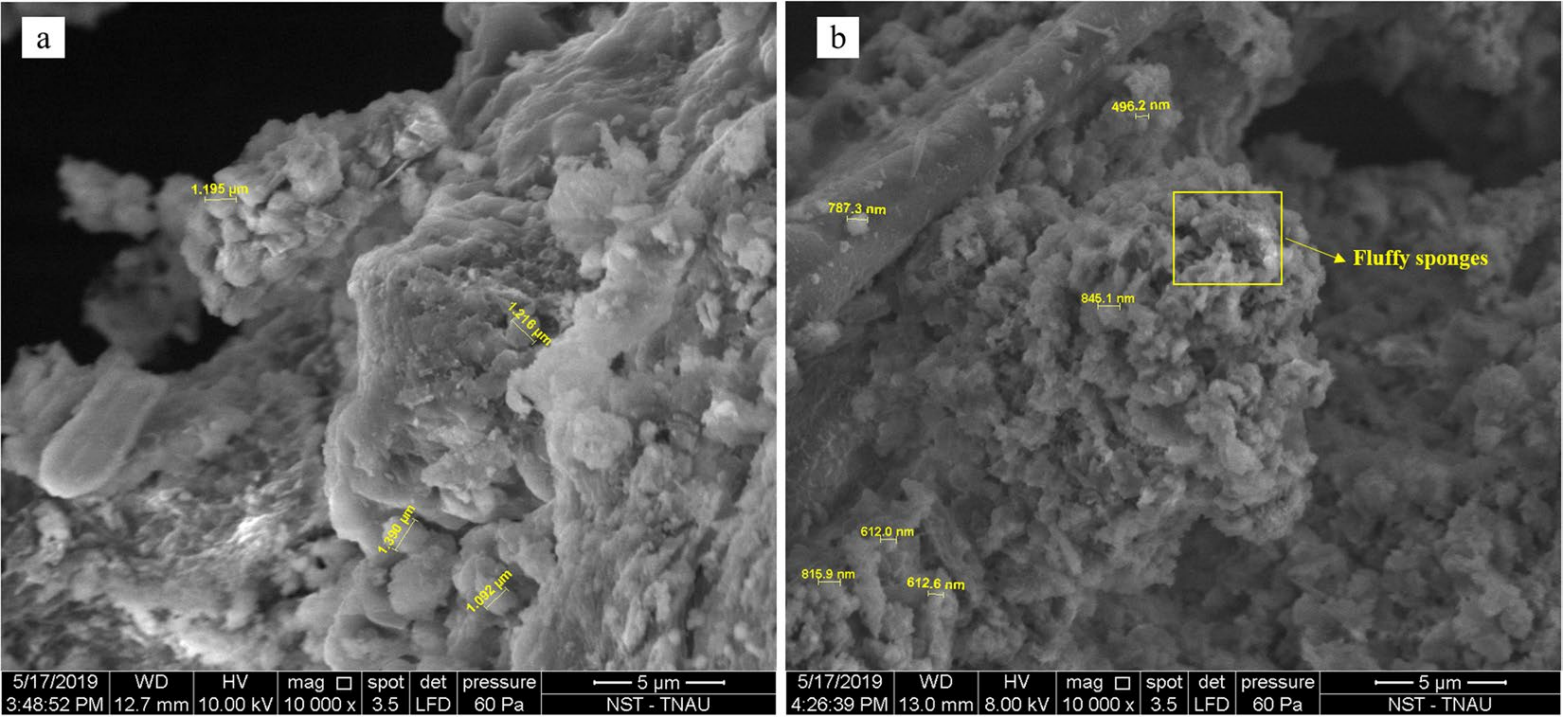
SEM micrographs of (**a**). PBM-ETPS and (**b**). PBM-ETPSH.

**Figure 8:**
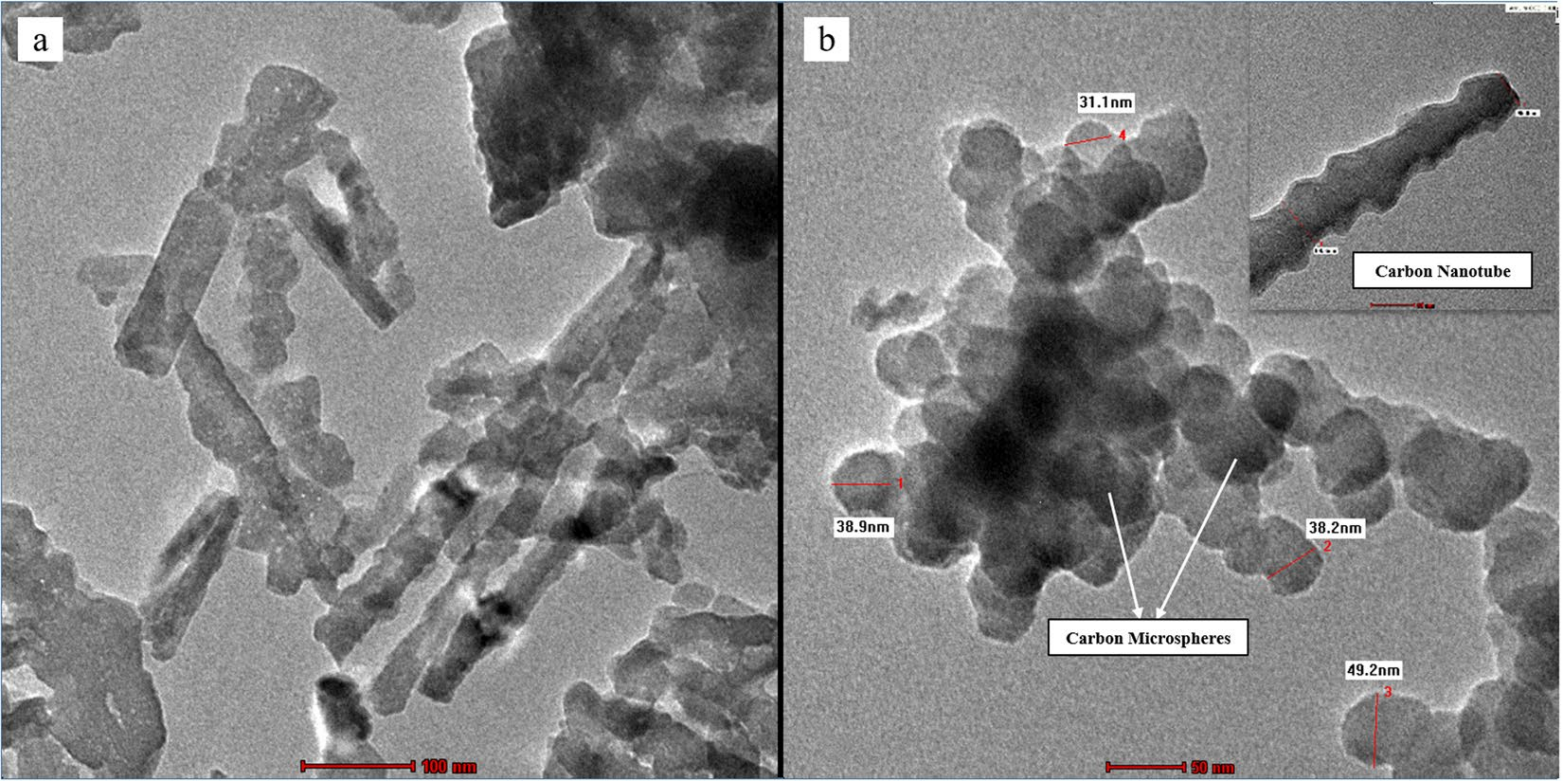
TEM micrographs of (**a**). PBM-ETPS and (**b**). PBM-ETPSH.

The scanning electron microscopy of PBM-ETPSH showed that it had formed a coarser surface as compared to smooth surface of PBM-ETPS with microspheres (Fig. 7). Small cavities, crevices, pores and rough surfaces on the hydrochar indicated the presence interconnected porous network of surfaces. These rugged surface formations were clearly visible from the micrographs wherein the size reduction of particles from µm (raw sludge) to nm (hydrochar) were also observed. Moreover, the surface of PBM-ETPSH exhibited particle dispersions in the form of fluffy sponges and spherically shaped particles with deeper fragmentation^4^. The internal surface modifications after the hydrothermal carbonization process was explored using transmission electron microscopy. The TEM micrographs (Fig. 8) depicted the formation of microspheres possessing a diameter ranging from 31.1 nm to 49.2 nm which was found to be an intensive size reduction than its precursor having a diameter of 100 nm. The irregular shaped precursor particles were converted to spherically shaped carbon materials. On the contrary, the formation of carbon nanotubes were also observed after the hydrothermal carbonization process.

### Fuel properties

**Figure 9:**
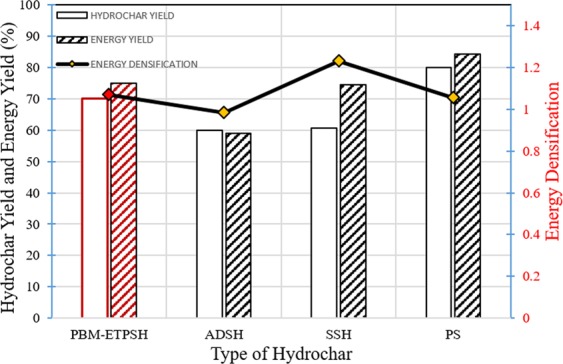
Fuel properties of different hydrochars.

**Figure 10:**
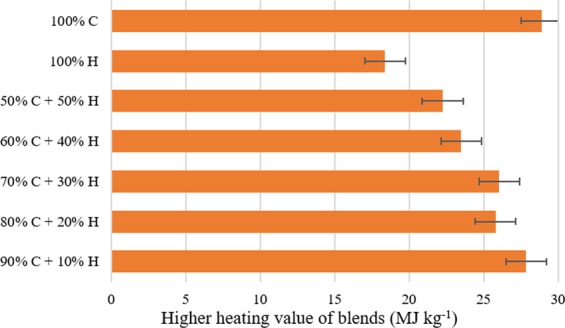
Higher heating value of optimized PBM-ETPSH and coal blends.

The HHV of PBM-ETPSH was more than PBM-ETPS by 6.7% with an energy densification value of 1.07. In a study, the hydrothermal carbonization of paper sludge resulted in the increment of higher heating value by 5.2% and energy densification up to 1.05^6^. Obviously, the higher yield of hydrochar with higher energy densification values enhanced the energetic recovery efficiency from hydrochar and a compound with higher heating value greater than 20 MJ kg^−1^ can be considered as a fuel^20^. The PBM-ETPSH of the current study obtained an energetic recovery efficiency of 75.03% which was found to be higher than anaerobic digested sludge derived hydrochar (59.1%)^5^ and sewage sludge derived hydrochar (74.5%)^4^. An appraisal of hydrochar yield, energy densification and energy yields of optimized hydrochar (PBM-ETPSH), sewage sludge derived hydrochar (SSH)^4^, anaerobic digested sludge derived hydrochar (ADSH)^5^, paper sludge derived hydrochar (PSH)^6^, are exhibited in the Fig. 9. The variation in the HHVs of coal and PBM-ETPSH:coal blends are given in Fig. 10. It was noticed that increase in the proportion of hydrochar with coal resulted in the gradual decrease of HHV. A blend of 50:50 showed better higher heating value (22.25 MJ kg^−1^) with just a slight increase in 90:10 blend (27.84 MJ kg^−1^).

### Thermo-gravimetric analyses of PBM-ETPSH:Coal blends

**Figure 11:**
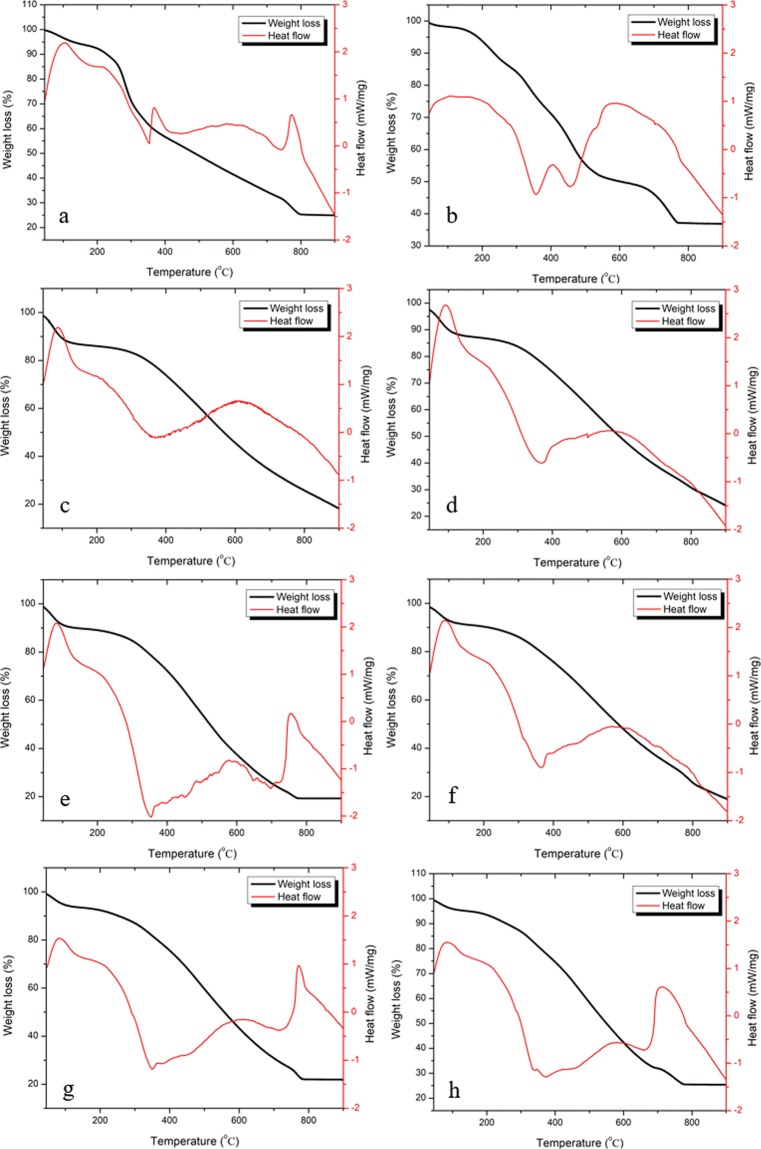
TGA/DSC curves of samples (**a**). Sludge, (**b**) Hydrochar, (**c**) Coal, (**d**) 90% coal + 10% hydrochar, (**e**) 80% coal + 20% hydrochar, (**f**) 70% coal + 30% hydrochar, (**g**) 60% coal + 40% hydrochar, (**h**) 50% coal + 50% hydrochar.

The effectiveness of co-combustion was assessed by simultaneous TGA/DSC analysis, wherein the weight loss due to the increase in temperature and the heat flow during combustion were determined. The TGA/DSC curves of the PBM-ETPS, PBM-ETPSH and the different blends are given in Fig. 11. The combustion behavior of PBM-ETPS significantly changed after HTC and the difference in peaks were observed due to the difference in reactivates of the sample components. The TGA curve of PBM-ETPS, PBM-ETPSH and the different blends showed two distinct mass loss regions. The first loss occurred from 100 °C to 200 °C due to the loss of water in the form of vapors from the sample and deterioration of long chained polymers into short chained hydrocarbons^21^. The DSC curve of this stage showed an endothermic peak in all samples due to the absorbance of heat by the water molecules in the samples. The second phase of loss corresponded to the volatilization of organic compounds and combustion of amorphous carbon that took place between 300–600 °C. The DSC curve of this second showed an exothermic peak due to the burning of amorphous carbon. This phase was rapid such that the slope falls exponentially compared to previous phase^22^. The final phase of combustion above 750 °C indicated the combustion of inorganic portions of the sludge and hydrochar samples. In other words, these materials corresponded to ash content of the sludge and hydrochar samples beyond which there was no further weight loss.

The thermogravimetric curves of the PBM-ETPSH:Coal blends exhibited similar weight loss curves. However, the weight percentage of hydrochar exhibited a relation over the curve formations. Due to the presence of moisture content, the thermogravimetric curve of coal exhibited a significant slope. However, on combination with hydrochar with lower moisture content, the curve corresponding to moisture loss decreased. This became evident at higher proportions of hydrochar in the blend wherein the curve corresponding to moisture loss became blunt. Similar reduction was observed during the co-combustion of lignite with coconut fiber and eucalyptus leaves derived hydrochars at different proportions^23^. After combustion, the weight loss percentage attained a constant line which depicted the proportion of ash content in the blends. The residual ash content of coal alone exhibited higher value (35%) while blending with PBM-ETPSH decreased the ash generated after combustion. Among the blends, lowest ash residue was obtained for 80:20 (20%) and highest ash residue was obtained for 50:50 (26%). Eventually, the ash residue should also be taken into account for the commercial blending of coal and PBM-ETPSH.

## Conclusion

The current study indicated that HTC of PBM-ETPS is an effective way of converting it into energy driven fuel. The proximate, ultimate analyses and the HHVs ascertained this. The blending of PBM-ETPSH with coal showed an HHV of 22.2 MJ kg^−1^ thus confirming the efficacy of PBM-ETPS derived wastes as an energy alternative. Further studies can be performed to reduce the process time for HTC and the commercial utilization of hydrochar as an energy source in industrial boilers.
